# High Red Cell Distribution Width Is Associated with Worse Prognosis in Early Colorectal Cancer after Curative Resection: A Propensity-Matched Analysis

**DOI:** 10.3390/cancers14040945

**Published:** 2022-02-14

**Authors:** Kung-Chuan Cheng, Yueh-Ming Lin, Chin-Chen Liu, Kuen-Lin Wu, Ko-Chao Lee

**Affiliations:** 1 Division of Colorectal Surgery, Department of Surgery, Kaohsiung Chang Gung Memorial Hospital and Chang Gung University College of Medicine, Kaohsiung 833, Taiwan; alcohol@cgmh.org.tw (Y.-M.L.); kunn913@cgmh.org.tw (K.-L.W.); kclee@cgmh.org.tw (K.-C.L.); 2 School of Medicine, Chung Shan Medical University, Taichung 406, Taiwan; 3 Department of Family Medicine, E-Da Dachang Hospital, Kaohsiung 833, Taiwan; ed106112@edah.org.tw

**Keywords:** red cell distribution width, colorectal cancer, prognosis

## Abstract

**Simple Summary:**

The association between red blood cell distribution width (RDW) and the prognosis of certain cancers has been established. However, RDW is also related to age, nutrition status, and many systemic disorders. Therefore, it is still unclear whether the association is contributed by these confounders. Here, we present a propensity-score match study demonstrating that RDW is an independently negative predictor for overall survival, disease-free survival, and cancer-specific survival in patients with stage I-II colorectal cancer.

**Abstract:**

The red blood cell distribution width (RDW) is a simple and widely available parameter obtained from a complete blood cell count test and is usually used in the analysis of anemia. Recently, studies have discovered the association between RDW and the host inflammatory response of cancer patients. We aimed to determine the prognostic value of RDW in colorectal cancer (CRC) patients. 5315 total patients with stage I-II CRC from the Chang Gung Memorial Hospital between 2001 and 2018 were enrolled. The study cohort was divided into two groups using RDW = 13.8 as the cutoff value as determined by receiver operating curve. High RDW had worse overall survival (OS), disease-free survival (DFS), and cancer-specific survival (CSS), and was also independently related to older age, more advanced tumor stage, lower albumin level, lower hemoglobin level, and more co-morbidities including diabetes, hypertension, and chronic kidney disease. We performed a propensity-score matched analysis to balance the heterogeneity between the two groups and to reduce the influence of confounding factors that may have compromised the prognosis. High RDW remained a negative predictor of OS (HR = 1.49, 95% CI: 1.25–1.78), as well as DFS and CSS. In conclusion, this is the first report using propensity matching to demonstrate the relationship between RDW and the prognosis of early-stage CRC patients.

## 1. Introduction

Colorectal cancer (CRC) is the third most common cancer worldwide, with more than 1 million new cases per year accounting for over 600,000 annual deaths. Moreover, the annual incidence is expected to increase in the next decade [1].

Patients with early-stage CRCs (i.e., stage I or II) are mainly treated with curative operation and are expected to have favorable survival. The tumor-node-metastatic (TNM) system is the most used predictor for prognosis of CRC patients after treatment. Other parameters commonly used as prognostic predictors include tumor location, clinicopathological findings such as lymphovascular invasion or perineural invasion, and serum carci-noembryonic antigen (CEA) level [2,3,4]. However, about 10–20% of patients still develop recurrent disease, which calls for the demand to search for other supplementary biomarkers in order to identify high-risk patients [5].

Red cell distribution width (RDW) is a parameter obtained from a complete blood cell count. It is a hematological parameter which measures the range for the volume and size of the RBC. Since RDW represents the variation of erythrocyte sizes, it is usually used traditionally to differentiate between types of anemias. Aside from its use in the study of anemia, the diagnostic value of RDW has been ignored in the past. In addition to its relationship with physical conditions such as age or pregnancy, increased RDW value has also been recently reported to be associated with several disorders including cardiovascular disease, diabetes mellitus, infectious disease, liver failure, or kidney failure [6]. Besides, more and more data have shown that this simple, easily-accessible, and inexpensive parameter may be a strong independent factor for prognosis, whether in the general population or in cancer patients [7].

Previous studies revealed that high RDW, in comparison with low RDW, may be a potential negative predictor for prognosis in CRC patients [8,9,10,11,12,13,14]. However, past results were limited by possible selection bias and confounding factors due to relatively small sample sizes and heterogeneity of baseline characteristics. Here, we present a multicenter retrospective study using a propensity-score matched analysis to determine the prognostic role of RDW in patents with stage I-II colorectal cancer. To the best of our knowledge, this is the largest study designed to reveal the correlation between RDW and the prognosis of CRC and is also the first report to focus on early-stage CRC.

## 2. Methods

### 2.1. Data Source

We conducted a retrospective analysis from the data retrieved from Chang Gung Research Database (CGRD), a dataset derived from the electronical medical records from Chang Gung Memorial Hospital (CGMH). CGMH is composed by two medical centers, two regional hospitals, and three district hospitals, and is the biggest hospital system in Taiwan. According to the cancer registry report from the Taiwan Ministry of Health and Welfare, CGMH provides approximately 14% of healthcare services for Taiwan’s cancer population [15]. CGRD is deidentified and is systematically updated annually for new data generated in CGMH. It contains clinical epidemiological data, laboratory test data, inpatient and outpatient data, emergency healthcare, pathological reports, disease category data, surgery data, and cancer registry data [16].

This study was approved by the institutional review board (IRB) of the Chang Gung Medical Foundation (approval number: 201901075B0). Since all patient’s data collected in this study were deidentified, the requirement for participants’ informed consent was waived. All study methods were performed in accordance with the relevant guidelines and regulations of IRB of Chang Gung Memorial Hospital.

### 2.2. Study Population

Patients diagnosed with stage I or II colorectal cancer who underwent curative surgery between January 2004 to April 2018 were enrolled in this study. Patients with positive lymph node (stage III), distant metastasis (stage IV), lack of pathological report of malignancy, or patients who lacked complete blood cell count data within one week prior to the operation were excluded.

### 2.3. Covariates

Baseline variables considered in the analyses included patient age, gender, tumor location, white blood cell (WBC) count, red blood cell (RBC) count, RDW, hemoglobin (Hb), platelet count, serum albumin level, and serum CEA level. Two RDW measurements were used in clinical practice, namely the RDW-coefficient of variation and the RDW-standard deviation. We used the coefficient of variation (presented as percentage) since it was more available in our database, and also since there was research which noted that RDW-coefficient of variation, compared with RDW-standard deviation, was a better predictor in the prognosis of colon cancer patients [8]. We also incorporated data on comorbidities including diabetes mellitus, hypertension, dyslipidemia, and chronic kidney diseases. The American Joint Committee on Cancer (AJCC) stages were originally coded in the database according to the 6th, 7th, or 8th AJCC edition, depending on the date of diagnosis, and related data were all transformed to staging according to the 8th AJCC edition to maintain consistency for the purpose of this study.

### 2.4. Outcomes

Our primary outcome was overall survival (OS) of patients diagnosed with stage I or II colorectal cancer who had undergone curative surgical resection for their primary tumor. The OS was defined from the date on which the patient underwent curative operation to the date of death by any cause. Secondary endpoints were cancer specific survival (CSS) and disease-free survival (DFS).

### 2.5. Statistics

Categorical variables were expressed as frequencies and percentages and continuous variables were expressed as mean with standard deviation (SD). Receiver operating curve (ROC) analysis was used to obtain the optimal cutoff value of RDW for death by any cause. Chi-squared test was used to compare categorical variables including sex, tumor location, stage, histologic grade, CEA level, and patient comorbidities such as diabetes mellitus, hypertension, chronic kidney disease, or dyslipidemia. As for continuous variables, the Mann-Whitney test or Student’s *t*-test were used for comparison depending on the result from the Kolmogorov-Smirnov test for normality. Kaplan-Meier curve analysis was used to estimate the cumulative OS, and log-rank test was used for statistical analysis of the difference between groups.

Propensity score matching (PSM) analysis was used to assure that there was no major difference in observed patients’ baseline characteristics (1:1 matching with greedy method). Standardized mean difference (SMD) was used to evaluate covariate balance after propensity matching.

All analyses were conducted using SAS (version 9.4; SAS Institute Inc., Cary, NC, USA), MedCalc (version 20; MedCalc Software Ltd., Ostend, Belgium), and NCSS Statistical Software (version 10; LLC, Kaysville, UT, USA).

## 3. Results

### 3.1. Patient Characteristics

From January 2004 to April 2018, 12,369 patients with colorectal cancer who had undergone radical resection for primary tumor were identified in the CGRD. Cases with carcinoma in situ (*n* = 412), stage III (*n* = 4266), stage IV (*n* = 1977), and incomplete record of pathological staging (*n* = 266) were excluded, and a total of 5448 stage I-II colorectal cancer patients were enrolled. Concerning those, patients who lacked preoperative complete blood count data within one week prior to the operation were excluded (*n* = 285). In conclusion, a total of 5135 stage I-II colorectal cancer patients who underwent radical resection of primary tumors were enrolled in this study and their data were analyzed retrospectively.

In our study cohort, the median age was 64 years old, 59.9% of patients were male, 48.9% were diagnosed with rectal cancer, and 40.6% were stage I disease. The median follow up duration was 5.4 years. The five-year recurrence rate was 3.8%(*n* = 79) for stage I patients and 10.5% (*n* = 320) for stage II patients. The five-year OS, DFS, and CCS were 83.8%, 80.4%, and 92.7%, respectively. Further details of patients’ baseline characteristics are listed in Table 1.

### 3.2. Unadjusted Survival

**Figure 1:**
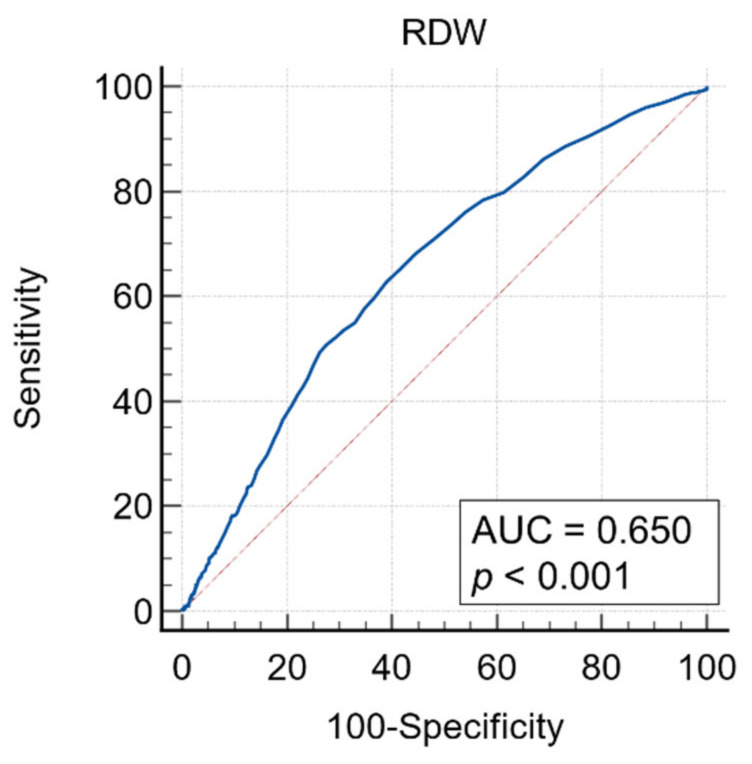
ROC analysis of RDW and five-year overall survival. Area under curve (AUC) was 0.65. Sensitivity and specificity were 62.7% and 61.0% at an optimal cutoff value 13.8.

The median of preoperative RDW was 13.6 (interquartile range (IQR): 12.9–14.9) for the entire cohort. ROC analysis for five-year OS indicated the area under curve was 0.65. Both the Youden Index method and the minimum distance from left-upper corner method showed the same optimal cutoff value of 13.8. Sensitivity and specificity were 62.7% and 61.0% using this cutoff value (Figure 1).

**Figure 2:**
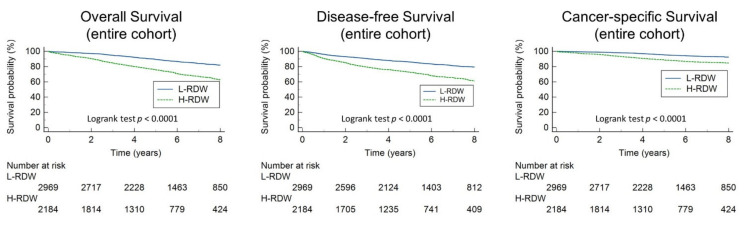

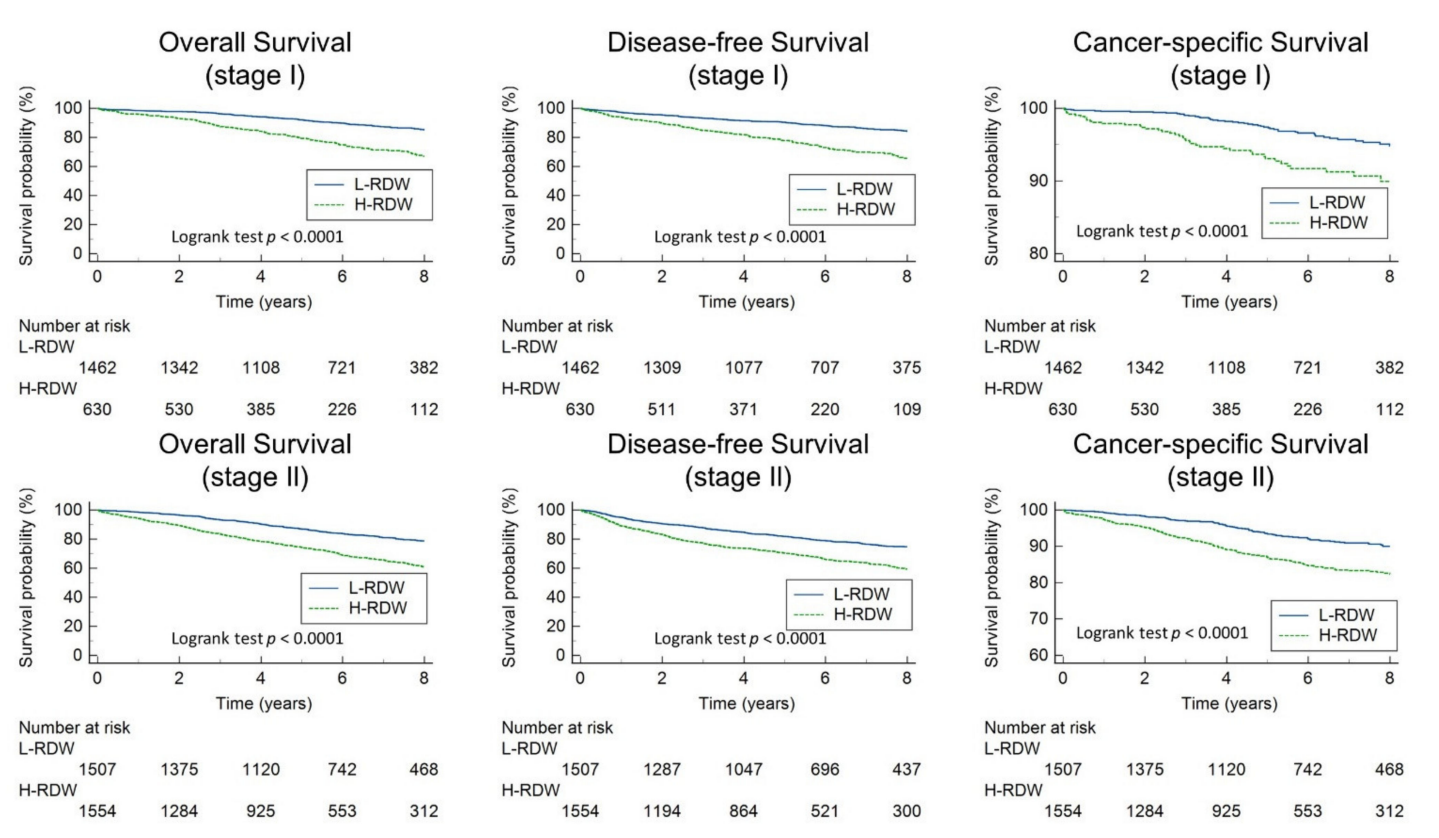
Overall survival, disease-free survival, and cancer-specific survival stratified by RDW in the entire cohort, stage I, or stage II patients.

We divided the patients into two groups according to their preoperative RDW value: the H-RDW group (RDW > 13.8, *n* = 2969) and the L-RDW group (RDW ≤ 13.8, *n* = 2184). Univariate analysis showed that the H-RDW group had worse OS (HR = 2.43, 95% CI: 2.15–2.75, *p* < 0.001), as well as worse DFS (HR = 2.18, 95% CI: 1.95–2.45, *p* < 0.001) and CSS rate (HR = 2.41, 95% CI: 1.97–2.93, *p* < 0.001), and this result was consistent for the respective analyses of stage I and stage II patients. Results are shown in Figure 2. Compared with the L-RDW group, H-RDW group tended to be older (median age 68 years vs. 63 years, *p* < 0.001), had more cases of stage II disease (71.2% vs. 50.8%, *p* < 0.001), had lower hemoglobin level (median 11.3 g/dL vs. 13.5 g/dL, *p* < 0.001), and had lower albumin level (median 3.9 g/dL vs. 4.3 g/dL, *p* < 0.001). The H-RDW group also had more patients with diabetes mellitus (24.6% vs. 20.6%, *p* < 0.001), hypertension (47.8% vs. 41.2%), and chronic kidney diseases (6% vs. 2.5%). The RDW values of stage I patients were significantly lower than those of stage II patients (13.3 vs. 13.9, *p* < 0.001). As for the tumor location, the RDW value was higher in patients with right-sided colon cancer than in patients with left-sided colon cancer in our study cohort (median 14.65 vs 13.6, *p* < 0.001). Other differences regarding patients’ characteristics and laboratory data between the two groups are listed in Table 2.

**Figure 3:**
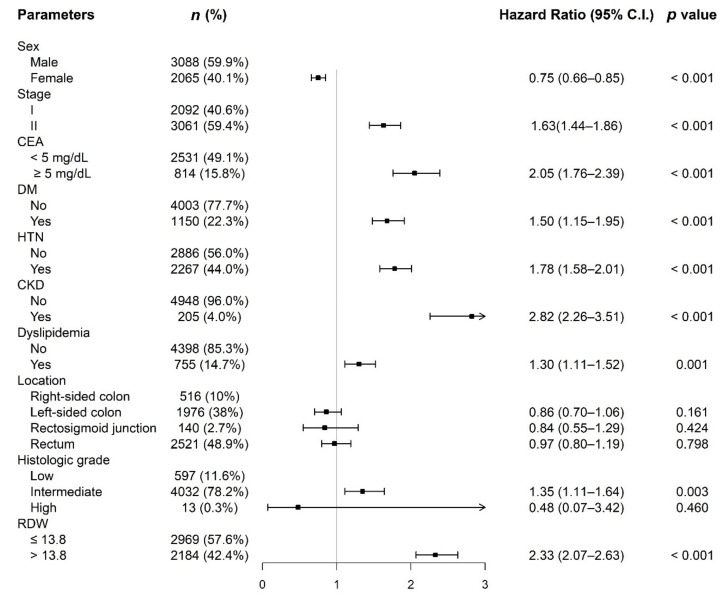
Hazard ratio of RDW, as well as the hazard ratios of other parameters in the unadjusted cohort.

Figure 3 shows the hazard ratio of RDW, as well as the hazard ratios of other parameters in the unadjusted cohort.

### 3.3. Propensity-Matched Analysis

PSM was performed to adjust for the heterogeneity between L-RDW and H-RDW groups. After 1:1 PSM, thirty-five percent of L-RDW group (*n* = 1050) and 48% of H-RDW group (*n* = 1050) were analyzed. The baseline characteristics were balanced in the matched groups (SMD < 0.1; *p* < 0.05), as presented in Table 2.

**Figure 4:**
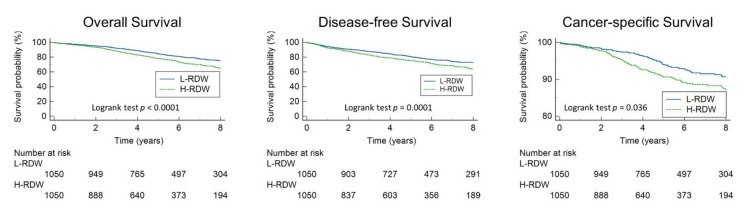
Overall survival, disease-free survival, and cancer-specific survival stratified by RDW after propensity-score matching.

After PSM, the H-RDW group still presented with significantly worse OS (HR = 1.49, 95% CI: 1.25–1.78, *p* < 0.001), DFS (HR = 1.39, 95% CI:1.18–1.64, *p* < 0.001), and CSS (HR = 1.37, 95% CI:1.02–1.85, *p* = 0.036), as shown in Figure 4.

**Figure 5:**
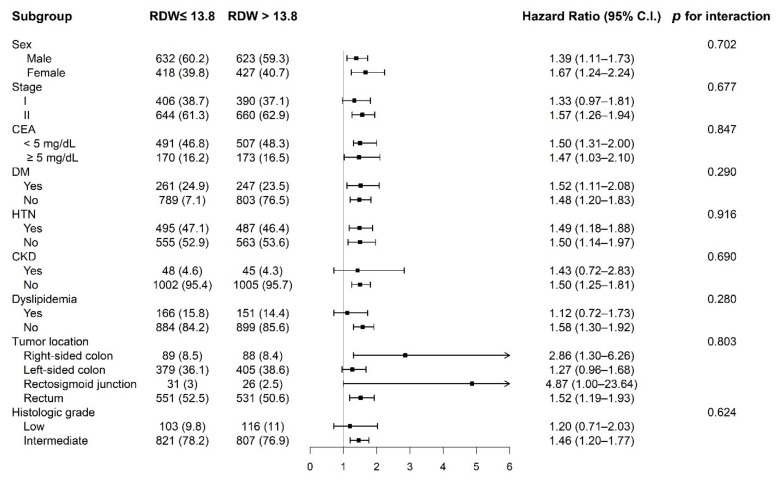
Stratified analyses of overall survival in the matched cohort.

Figure 5 presents the stratified analyses of OS in the matched cohort. There was no significant heterogeneity in HR, and favorable survival rate associated with low RDW was consistent across all subgroups.

## 4. Discussion

To the best of our knowledge, this is the largest study to demonstrate the correlation between RDW and the prognosis of CRC. Our study showed that high preoperative RDW was a negative predictive factor for the survival of patients with stage I or II CRC.

It is well-known that malignant tumors induce chronic inflammation and malnutrition status [6,17], which further compromises the patients’ quality of life and life expectancy. Several studies reported that in cancer or inflammatory diseases, RDW level was closely related to proinflammatory cytokines such as interleukin-6, tumor necrosis factor alpha, and cytokeratin 19 fragment, and inflammatory biomarkers including erythrocyte sedimentation rate (ESR) and C-reactive Protein (CRP) [18,19,20]. The chronic inflammation evoked by cancer leads to inadequate production of erythropoietin, suppression of erythropoiesis, malnutrition, increased oxidative stress, and cachexia [21]. These findings imply that increased RDW can reflect inflammatory response, malnutrition status, and elevated oxidative stress, thus leading to the hypothesis that high RDW is associated with worse prognosis.

Several studies showed the relationship between high RDW value with worse prognosis in CRC patients [8,9,10,11]. However, it remained questionable whether this relationship was specific for any subgroup of patients. Zhang et al. reported a retrospective study of 625 non-metastatic rectal patients [8]. In their multivariate analysis, high RDW was related to poor DFS. Similarly, Li et al. reported that increased RDW was associated with shorter DFS and OS in a study which enrolled 271 patients [9]. Neither study demonstrated whether the trend was consistent for patients in all cancer stages within their study population. In a retrospective study of 240 patients, Han et al. reported that high RDW was an independent negative predictor of DFS and OS for metastatic CRC, but not for non-metastatic CRC [10]. On the other hand, in a retrospective study which included 591 patients, Pedrazzani et al. reported that high RDW was only related to OS but not DFS in CRC [11]. In their study, they found that the difference was only demonstrated until after greater than five-years of follow-up, and it seemed that this relationship was particular to stage I CRC. After multivariate analysis in which factors of age, tumor stage, and tumor location were controlled for, they could not prove high RDW to be an independent predictor for survival. In addition to its association with older age, an increased RDW may also be related to malnutrition, chronic metabolic diseases, or cardiovascular diseases, as previously described. However, most studies did not collect related data such as albumin level or comorbidities such as diabetes or hypertension. Therefore, some potential confounding factors that were not discovered could have compromised the data on CRC patient survival.

In this study, we enrolled patients with stage I or II colorectal cancer who had undergone curative operation. Using 13.8 as the cutoff point as determined by ROC, we demonstrated that the group with higher RDW had worse OS, DFS, and CSS, and the difference was evident even in the early stages of follow-up. As with the other studies [7,8,9,10], the high RDW group tended to be older, had more advanced tumor invasion (T stage), had lower hemoglobin level, and higher CEA level. Besides, the high RDW population also had significantly lower albumin level and increased rate of comorbidities with diabetes, hypertension, and chronic kidney disease.

Recently, the relations between progression and the tumor location of CRC have been discussed. Some may ask the question whether the primary tumor location may affect RDW level. In our study cohort, compared to patients with left-sided colon cancer, those with right-sided colon cancer had higher RDW level. This finding is consistent with the study by Fancellu et al. [22]. However, this difference was not observed after propensity-score matching, which indicates that there may have been other confounding factors contributing to the difference of RDW level between those with right-sided and left-sided colon cancer.

Since high RDW was closely related to other negative predictors for a patient’s survival, it was difficult to distinguish whether it alone could be an independent predictor for survival, or if it was simply a reflection of the other confounding factors. Hence, we performed a propensity-matched analysis, a tool to adjust group effect for measured confounders in nonrandomized studies. After matching, high RDW was confirmed to be a strong negative predictor for OS, DFS, and CSS in early colorectal cancer. While curative operation is the mainstay treatment for early colorectal cancer, the role of adjuvant chemotherapy is significantly more important regarding high-risk stage II patients. Currently, factors that define a patient as “high-risk” include the following: T4 tumor, poorly differentiated histology, inadequate number of lymph nodes analyzed, presentation of lymphovascular invasion or perineural invasion, tumor perforation, and luminal obstruction [23]. RDW, as a prognostic predictor related to cancer-induced inflammatory response, might provide some information for the decision of adjuvant therapy. By utilizing the combination of RDW and other serum tumor markers specific to gastrointestinal cancers such as CEA or CA19-9, we might be able to improve the prognosis of early-stage CRC patients.

To our knowledge, among all the studies that discussed the topic of RDW as a prognostic factor in CRC, our study had the largest sample size. This was also the first study to use propensity-matched analysis to balance the differences in baseline characteristics between groups. However, there remained some potential limitations in this study. First, though propensity matching was able to generate balanced groups, potential selection bias still existed due to the nature of retrospective study. Second, due to the limitations of CGRD, pathological factors such as lymphovascular invasion, perineural invasion, microsatellite instability, tumor size, or resection margin could not be acquired in our study, and these factors were known to be related to the prognosis of CRC [24,25,26]. In ROC analysis, we used death by any cause to determine the cutoff value of RDW. The cutoff value may be different if other events, such as recurrence of the disease, were used for analysis. Therefore, further research is required to investigate which cutoff value is suitable for clinical use of RDW in CRC patients. In this study, we used a single, pre-treatment blood test of RDW as a predictor of the prognosis and recurrence. While it seems like RDW value may vary over time, it is also interesting to question whether it can be used to monitor CRC after radical resection. A prospective study will be needed to examine confounders and to further clarify the prognostic value of RDW.

## 5. Conclusions

Preoperative RDW is a simple and widely available blood parameter which is strongly and independently associated with the OS, DFS, and CSS of patients with early-stage colorectal cancer undergoing curative operative intervention. It might be used as a supplementary biomarker for decision-making regarding surveillance and adjuvant therapy.

## Figures and Tables

**Table 1:** Baseline characteristics.

Characteristics	*n*
Age (median (IQR))	64 (56–74)
Sex (%)	
Male	3088 (59.9%)
Female	2065 (40.1%)
Tumor Location (%)	
Right-sided colon	516 (10%)
Left-sided colon	1976 (38%)
Rectosigmoid	140 (2.7%)
Rectum	2521 (48.9%)
Stage (%)	
I	2092 (40.6%)
II	3061 (59.4%)
Histologic Grade (%)	
Well	597 (11.6%)
Moderate	4032 (78.2%)
Poorly	13 (0.3%)
Unknown	511 (9.9%)
WBC (median (IQR))	6.7 (5.5–8.3)
RBC (median (IQR))	4.43 (4.03–4.77)
Hb (median (IQR))	12.7 (11.2–13.9)
Platelet (median (IQR))	241 (196–296)
RDW (median (IQR))	13.6 (12.9–14.9)
Albumin (median (IQR))	4.15 (3.8–4.4)
CEA level (%)	
Normal (<5)	2531 (49.1%)
Elevated (≥5)	814 (15.8%)
Unknown	1808 (35.1%)
Diabetes Mellitus (%)	
Yes	1150 (22.3%)
No	4003 (77.7%)
Hypertension (%)	
Yes	2267 (44.0%)
No	2886 (56.0%)
CKD (%)	
Yes	205 (4.0%)
No	4948 (96.0%)
Dyslipidemia (%)	
Yes	755 (14.7%)
No	4398 (85.3%)

**Table 2:** Characteristics between groups before and after propensity matching.

Characteristics	Entire Cohort				Propensity-Matched Cohort			
	L-RDW (≤13.8) *n* = 2969	H-RDW (>13.8) *n* = 2184	*p*	SMD	L-RDW (≤13.8) *n* = 1050	H-RDW (>13.8) *n* = 1050	*p*	SMD
Age (median (IQR))	63 (55–75)	68 (57–77)	<0.001	0.244	66 (57–74)	66 (57–76)	0.321	0.026
Gender (%)			0.886	0.005			0.689	0.017
Male	1782.0 (60.0)	1306.0 (59.8)			632 (60.2)	623 (59.3)		
Female	1187 (40.0)	878 (40.2)			418 (39.8)	427 (40.7)		
Tumor Location (%)			<0.001	0.264			0.642	0.057
Right-sided colon	200 (6.7)	316 (14.5)			89 (8.5)	88 (8.4)		
Left-sided colon	1138 (38.3)	838 (38.4)			379 (36.1)	405 (38.6)		
Rectosigmoid	82.0 (2.8)	58.0 (2.7)			31 (3)	26 (2.5)		
Rectum	1549.0 (52.2)	972.0 (44.5)			551 (52.5)	531 (50.6)		
Stage (%)			<0.001	0.428			0.470	0.031
I	1462 (49.2)	630 (28.8)			406 (38.7)	390 (37.1)		
II	1507.0 (50.8)	1554.0 (71.2)			644 (61.3)	660 (62.9)		
Histologic Grade (%)			<0.001	0.142			0.826	0.041
Well	365 (13)	212 (9.7)			103 (9.8)	116 (11)		
Moderate	2321 (78.2)	1711 (78.3)			821 (78.2)	807 (76.9)		
Poorly	4 (0.1)	9 (0.4)			3 (0.3)	3 (0.3		
Unknown	259 (8.7)	252 (11.5)			123 (11.7)	124 (11.8)		
WBC (median (IQR))	6.6 (5.6–8.0)	6.8 (5.5–8.7)	<0.001	0.187	6.55 (5.5–8.1)	6.6 (5.3–8.3)	0.554	0.008
RBC (median (IQR))	4.5 (4.17–4.79)	4.26 (3.85–4.73)	<0.001	0.238	4.31 (3.93–4.64)	4.27 (3.87–4.69)	0.641	0.035
Hb (median (IQR))	13.5 (12.4–14.5)	11.3 (10.1–12.7)	<0.001	1.163	12.4 (11.5–13.6)	12.4 (11.3–13.5)	0.353	0.026
Platelet (median (IQR))	232 (194–275)	258 (199–336)	<0.001	0.440	238 (200–286)	236 (189–295)	0.377	0.001
Albumin (median (IQR))	4.3 (4.0–4.5)	3.9 (3.5–4.26)	<0.001	0.779	4.1 (3.7–4.31)	4.1 (3.77–4.31)	0.929	0.006
CEA (%)			<0.001	0.169			0.684	0.0.38
Normal	1511.0 (50.9)	1020.0 (46.7)			491 (46.8)	507 (48.3)		
Elevated	391.0 (13.2)	423.0 (19.4)			170 (16.2)	173 (16.5)		
Unknown	1067.0 (35.9)	741.0 (33.9)			389 (37.0)	370 (35.2)		
Diabetes Mellitus (%)			<0.001	0.094			0.476	0.031
Yes	613.0 (20.6)	537.0 (24.6)			261 (24.9)	247 (23.5)		
No	2356 (79.4%)	1647 (75.4)			789 (7.1)	803 (76.5)		
Hypertension (%)			<0.001	0.132			0.727	0.015
Yes	1224.0 (41.2)	1043.0 (47.8)			495 (47.1)	487 (46.4)		
No	1745 (58.8)	1141 (52.2)			555 (52.9)	563 (53.6)		
CKD (%)			<0.001	0.178			0.750	0.014
Yes	73.0 (2.5)	132.0 (6.0)			48 (4.6)	45 (4.3)		
No	2962 (97.5)	2052 (94.0)			1002 (95.4)	1005 (95.7)		
Dyslipidemia (%)			0.750	0.009				0.040
Yes	439.0 (14.8)	316.0 (14.5)			166 (15.8)	151 (14.4)		
No	2530 (85.2)	1868 (85.5)			884 (84.2)	899 (85.6)		

SMD, standardized mean difference.

## Data Availability

Data can be provided by the corresponding author upon reasonable request.
